# Effect of dsDNA binding to SmD-derived peptides on clinical accuracy in the diagnosis of systemic lupus erythematosus

**DOI:** 10.1186/ar2266

**Published:** 2007-07-18

**Authors:** Michael Mahler, Aderajew Waka, F Hiepe, Marvin J Fritzler

**Affiliations:** 1 Development and Production, Dr Fooke Laboratorien, Mainstraße 85, Neuss 41469, Germany; 2 Charité-University of Medicine Berlin, Internal Medicine Department of Rheumatology and Clinical Immunology & German Rheumatism Research Centre of Berlin, Department of Autoimmunology, Charitéplatz 1, 10117 Berlin, Germany; 3 Medical Clinic for Rheumatology and Clinical Immunology, Charité – Universitätsmedizin Berlin, Charitéplatz 1, 10117 Berlin, Germany; 4 Department of Medicine and Biochemistry & Molecular Biology, Faculty of Medicine, University of Calgary, 3330 Hospital Dr. NW, Calgary T2N 4N1, Canada

## Abstract

Systemic lupus erythematosus is characterized by antibodies to a variety of intracellular self-antigens, such as dsDNA and Sm, and these serve as hallmarks in the diagnosis of systemic autoimmune diseases. Several studies have shown that SmD1 and SmD3 synthetic peptides represent highly functional antigens for autoantibody detection and thus for diagnostic applications. The present study analysed the technical and clinical accuracy of an anti-SmD1 (amino acids 83–119) and an anti-SmD3 (amino acids 108–122) ELISA for the detection of anti-Sm antibodies. Depending on the cut-off value of the SmD1 ELISA, we found a high degree of concordance between the two tests. At an optimized cut-off value of 100 units for SmD1 we found the same clinical sensitivity (12.5%) and specificity (100%) in a group of systemic lupus erythematosus patients (*n *= 48) and in controls (*n *= 99). The concordance at this cut-off value was 100% (*P *< 0.0001; χ^2 ^= 127.61). Using a second panel of sera (*n *= 65) preselected based on positive anti-Sm results, we confirmed the high degree of concordance between the two assays. Using dsDNA-coated ELISA plates and biotinylated peptides we confirmed the high dsDNA binding properties for SmD1, which were significantly higher than the SmD3-derived peptide. However, no cross-linking of anti-dsDNA antibodies to SmD1 was observed after adding increasing amounts of dsDNA to anti-dsDNA positive, anti-SmD1 negative serum. We therefore conclude that the reported difference in the sensitivity is related to the different cut-off levels and not to the detection of anti-dsDNA antibodies bridged via dsDNA to the SmD1 peptide. Moreover, we found that a subpopulation of anti-Sm antibodies cross-reacted with SmD1 and SmD3. Taken together, the data indicate that both SmD peptide ELISAs represent accurate assays and may be used as important standards for the detection of anti-Sm antibodies.

## Introduction

Systemic rheumatic diseases are characterized by circulating autoantibodies to more than 200 autoantigens, which can precede the clinical onset of the disease and thus have high prognostic value [1,2]. Among the earliest identified autoantibodies were those directed to components of U2–U6 small nuclear ribonucleoproteins (RNPs) known collectively as Sm, which are highly specific for systemic lupus erythematosus (SLE) [3]. Anti-Sm antibodies have therefore been included as one of the SLE classification criteria of the American College of Rheumatology [4].

The Sm antigen is part of the spliceosomal complex that catalyses the splicing of nuclear pre-mRNA and is composed of at least nine different polypeptides with molecular weights ranging from 9 to 29.5 kDa (SmB1, SmB', SmB3, SmD1, SmD2, SmD3, SmE, SmF and SmG) [5,6]. All of these core proteins, but most frequently the SmB and SmD polypeptides, are targets of the anti-Sm autoimmune response [3]. Since SmBB' and U1-specific RNPs share the cross-reactive epitope motif PPPGMRPP, SmD is regarded as the most SLE-specific Sm antigen [7]. Within the SmD autoantigen family, reactivity with SmD1/D3 is at least four times more common than SmD1/SmD2/SmD3 recognition, with a pronounced immunoreactivity to SmD1 [8]. In epitope-mapping studies of SmD1 and SmBB', the major reactivity was predominantly found in the C-terminal regions [9-17]. Small nuclear RNPs such as SmD1, SmD3, and SmBB' were recently shown to contain symmetrical dimethylarginine (sDMA), and these modified residues were shown to constitute major epitopes on the SmB and SmD polypeptides [14,18].

Anti-Sm reactivity is found in 5–30% of patients with SLE, and this frequency varies depending on the detection system, the selection criteria for study cohorts and the ethnicity of the SLE population under investigation [14-19]. Several immunoassays designed for research studies, as well as for diagnostic laboratory use, have been developed. The antigenic analytes employed in these tests included purified native proteins, recombinant polypeptides or synthetic peptides [14-22]. In independent studies, a high degree of clinical accuracy has been reported for SmD-derived peptide-based immunoassays (SmD1^83–119 ^and SmD3^108–122^) [14-16,20]. The SmD1 peptide has been shown to be dependent on casein as a cofactor for antibody binding, and the SmD3 peptide contains an sDMA residue as a key amino acid [14,23].

The present study was designed to evaluate two SmD peptide-based immunoassays and to analyse the putative effect of dsDNA/SmD peptide complex formation on the diagnostic accuracy of the SmD assays.

## Materials and methods

### Serum samples

A panel of sera (panel I) was collected from SLE patients (*n *= 48) and from patients with various control diseases including rheumatoid arthritis (*n *= 50), mixed connective tissue disease (MCTD) (*n *= 16), scleroderma (systemic sclerosis) (*n *= 17), polymyositis/dermatomyositis (*n *= 11), and other autoimmune disorders (*n *= 15). All samples were used in a previous study and were classified according to published criteria for each disease [16]. Sera were stored in aliquots at -80°C until use and were shipped on dry ice. None of the samples had more than two freezing and thawing cycles.

A second panel (panel II) of sera (*n *= 65) was selected based on a positive anti-Sm test in the QUANTA Plex 8™ addressable laser bead immunoassay (see below). The international antinuclear antibodies reference serum panel available from the Centre of Disease Control and Prevention (CDC, Atlanta, GA, USA) was also tested in the SmD peptide ELISAs [24].

Finally, a third panel of serum samples (panel III, *n *= 200) was collected at the Charité – Universitätsmedizin (Berlin, Germany), including samples from SLE patients (*n *= 100), from patients with infectious diseases (malaria, hepatitis B virus, hepatitis C virus, human immunodeficiency virus, five from each group; *n *= 20), from MCTD patients (*n *= 7), from CREST syndrome (calcinosis, Raynaud phenomenon, oesophageal dysmotility, sclerodactyly, and telangiectasia) patients (*n *= 8), from scleroderma patients (*n *= 10), from polymyositis patients (*n *= 6), from primary Sjögren's syndrome patients (*n *= 7), from rheumatoid arthritis patients (*n *= 22) and from normal controls (*n *= 20). The samples were used to validate the newly defined cut-off value of the SmD1 ELISA.

### Synthetic peptides

Synthetic peptides (SmD1, SmD3, PM1-α and Ribosomal P) were synthesized according to the Fmoc-chemistry at the Peptide Specialty Laboratories GmbH (PSL, Heidelberg, Germany) as previously described [14,15,25,26]. In brief, crude extract was purified by high-performance liquid chromatography. The quality and purity of the peptide were assessed by mass spectrometry and by analytical high-performance liquid chromatography.

### Anti-Sm antibody assays

#### Varelisa^® ^Sm antibodies

The Varelisa^® ^Sm assay (reference 18296; Phadia GmbH, Freiburg, Germany) is based on a recently identified peptide derived from the SmD3 sequence [14]. The SmD3 peptide comprises the 16 amino acids 108–122 of SmD3 (^108^AARG sDMA GRGMGRGNIF^122^) with an additional cysteine at the C-terminus and a sDMA residue at position 112.

#### Imtec-SmD1 antibodies

The Imtec-SmD1 ELISA (catalogue number IgG TC 60029; Human GmbH, Wiesbaden, Germany) is based on a synthetic peptide representing the C-terminal region of SmD1 (amino acids 83–119) first described by Riemekasten and colleagues in 1998 [15].

#### RNP/Sm ELISA

The RNP/Sm ELISA (catalogue number 25011; Dr Fooke Laboratorien GmbH, Neuss, Germany) is based on native highly purified RNP/Sm antigen containing U1–68 kDa, U1-A, U1-C, SmB, SmB', SmD1, SmD2, SmD3, SmE, SmF and SmG.

#### Sm ELISA

The Sm ELISA (catalogue number 25010; Dr Fooke Laboratorien GmbH) is based on native highly purified SmD antigen from a bovine source.

#### Addressable laser bead assay

Microspheres embedded with laser reactive dyes (Luminex Corporation, Austin, TX, USA) that were coupled to native Sm antigen were part of a commercial kit (QUANTA Plex 8™; INOVA Diagnostics Inc., San Diego, CA, USA). This profile test also allows for the semiquantitative detection of autoantibodies to chromatin, Jo-1, Rib-P, RNP, Scl-70, SS-A (Ro) and SS-B (La). The assay was performed according to the manufacturer's instructions as previously described [16,27].

#### dsDNA ELISA and native DNA indirect immunofluorescence

The dsDNA ELISA (catalogue number 25004; Dr Fooke Laboratorien GmbH) based on a recombinant plasmid DNA was used to measure anti-dsDNA antibodies. The assay was carried out according to the manufacturer's instructions for use. Anti-dsDNA reactivity (to native DNA) was confirmed using the slide test with *Crithidia luciliae *as the substrate (Fluorescent nDNA; ImmunoConcepts, Sacramento, CA, USA).

### Competive ELISA

To analyse the populations of anti-Sm antibodies contained in SLE sera, competitive ELISAs were carried out. Synthetic SmD1 and SmD3 peptides were serially diluted in sample buffer (Varelisa^® ^kit component), resulting in peptide concentrations from 1.5 to 100 μg/ml. As a negative control, dsDNA and recombinant ribosomal P2 protein were similarly diluted in sample buffer. The binding of the anti-Sm antibodies to SmD-coated ELISA plates was competed by a 30-minute preincubation at room temperature with the respective competitor peptide. Following the preincubation phase, the samples were transferred onto the ELISA plates and the assays were carried out according to the standard manufacturer's protocol of the Varelisa^® ^system. The percentage inhibition was calculated: (OD_without inhibitor _- OD_with inhibitor_)/OD_without inhibitor _× 100, where OD represents the optical density.

#### SmD/dsDNA binding experiments

Binding of SmD-derived peptides to dsDNA was studied on dsDNA-coated ELISA plates (Dr Fooke Laboratories). Soluble, biotinylated peptides (SmD1, SmD3, PM1-α and Ribosomal P) were serially diluted in dilution buffer, starting at concentrations of 1,000 ng/ml. Then 100 μl of the respective dilutions were added to the wells of dsDNA-coated ELISA plates and incubated for 30 minutes at room temperature. Unbound peptides were removed by three washing cycles with 350 μl washing buffer (kit component of the dsDNA ELISA) per well. Peptides able to bind to dsDNA, and therefore immobilized in the microtitre surface, were detected by streptavidin–horseradish peroxidase conjugate (KPL, Gaithersburg, MD, USA) at a concentration of 0.5 μg/ml in combination with 3,3',5,5'-tetramethylbenzidine (kit component of the dsDNA ELISA). The reaction was terminated with stop solution and the optical density was measured photometrically at 450 nm. The inhibitory effect of the SmD1 and SmD3 peptides was analysed by testing an anti-dsDNA-positive sample from a SLE patient on dsDNA-coated microtitre strips preincubated with increasing concentrations of the SmD peptides as described above. Detection of bound human anti-dsDNA antibodies was according to the instructions for use of the dsDNA ELISA (Dr Fooke Laboratories).

#### Bridging experiment

A serum sample with high-titre anti-dsDNA antibodies but no anti-SmD1 (amino acids 89–119) reactivity was spiked with increasing concentrations of dsDNA (0.4–100 μg/ml plasmid DNA; also used in the dsDNA ELISA; Dr Fooke Laboratories) and was incubated for 30 minutes at room temperature. The dilution series was subsequently tested for anti-dsDNA and anti-SmD1 reactivity in the ELISA according to the instructions for use of the respective kit.

### Statistical evaluation of results

The results obtained from the comparative study were evaluated using the Analyse-it Software (version 1.62; Analyse-it Software, Ltd, Leeds, UK). Receiver-operating characteristic curves, positive and negative predictive values as well as the clinical efficiency were calculated for each anti-Sm antibody assay. The Fisher exact test and the chi-squared test were used to analyse the statistical relevance of correlation between two proportions.

## Results

### Comparison of clinical accuracy of the SmD peptide ELISAs

Sera from 48 unselected SLE patients and from various control samples (*n *= 99) were tested by two different Sm autoantibody ELISAs (Varelisa^®^, Phadia GmbH; and Imtec-SmD1; Human GmbH). At the cut-off value of 25 units suggested by the manufacturer, 22/48 (45.8%) SLE sera and 22/99 (22.2%) controls were positive for anti-SmD1 antibodies (see Table 1). In contrast, 6/48 (12.5%) SLE sera but none of the control sera had antibodies to the SmD3-derived peptide.

**Table 1:** Overview of the assay performance of anti-SmD1 and anti-SmD3 ELISAs determined in independent studies

Disease/control group	SmD1 ELISA						SmD3 ELISA	
	
	Previous studies		Present study					Present study, panel I
			
	Riemekasten and colleagues, 1998 [15]	Jaekel and colleagues, 2001 [20]	Panel I (25 units)	Panel I (100 units)	Panel III (25 units)	Panel III (100 units)	Mahler and colleagues, 2005 [14]	
Systemic lupus erythematosus (*n*)	167	111	48	48	100	100	176	48
Controls (*n*)	372	144	99	99	100	100	449	99
Primary Sjögren syndrome (*n*)	15	10	-	-	7	7	24	-
Mixed connective tissue disease (*n*)	23	13	16	16	7	7	26	16
Rheumatoid arthritis (*n*)	28	10	50	50	22	22	86	50
Miscellaneous (*n*)	73	21	15	15	5	5	21	15
Undifferentiated connective tissue disease (*n*)	-	22	-	-	-	-	-	-
Scleroderma (*n*)	20	11	17	17	18	18	26	17
Normal human donor (*n*)	105	50	-	-	20	20	192	-
Polymyositis scleroderma overlap syndrome (*n*)	-	7	11	11	6	6	-	11
Human immunodeficiency virus (*n*)	88	-	-	-	5	5	-	-
Hepatitis B virus (*n*)	20	-	-	-	5	5	-	-
Hepatitis C virus (*n*)	-	-	-	-	5	5	30	-
Cytomagalovirus (*n*)	-	-	-	-	-	-	22	-
Epstein–Barr virus (*n*)	-	-	-	-	-	-	25	-
Sensitivity (%)	70	36	45.8	12.5	47	21	15.9	12.5
Specificity (%)	91.7	97.2	77.8	100	84	100	99.8	100

**Figure 1:**
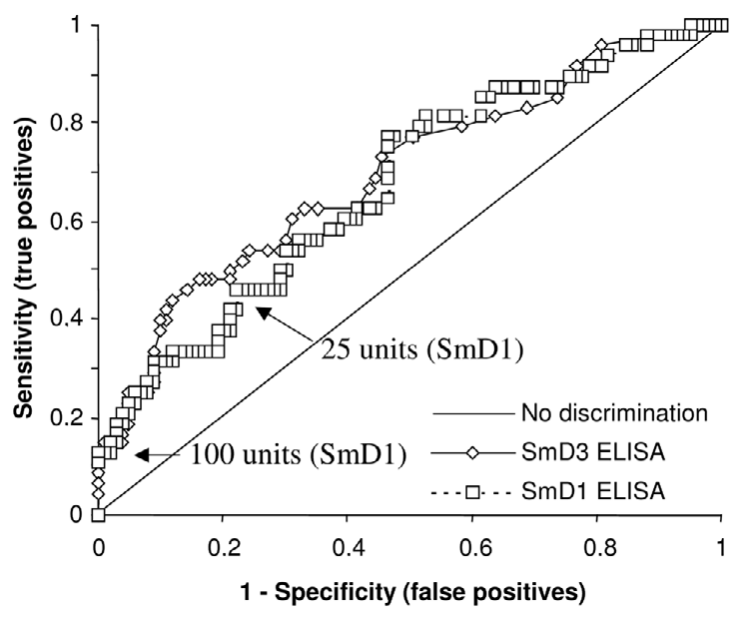
Receiver-operating characteristic analysis of two SmD peptide-based anti-Sm antibody assays. The results of this comparative study were used to generate receiver-operating characteristic curves. The discrimination between systemic lupus erythematosus patient samples and control samples was similar for both SmD immunoassays.

To compare the ability of both assays to differentiate SLE patients from various controls, a receiver-operating characteristic analysis was performed. Both assays showed a comparable differentiation between SLE patients and controls as revealed by the area under the curve of the receiver-operating characteristic analysis (see Figure 1). After adjusting the cut-off value of the SmD1 immunoassay to 100 IU/ml to achieve 100% specificity, the same sensitivity (12.5%) was found as in the SmD3 peptide-based ELISA (see Tables 1 and 2). At cut-off values of 100 units for SmD1 and of 15 U/ml for SmD3, the agreement was 100% (*P *< 0.0001; χ^2 ^= 127.61).

**Table 2:** Overview of samples >25 units in the SmD1 (amino acids 83–119) ELISA

Sample ID	Diagnosis	SmD3 (U/ml)	SmD1 (U/ml)
25413	SLE	2.8	27.50
25414	SLE	3.9	29.40
25415	SLE	2.9	26.40
25419	SLE	8.9	91.70
25423	SLE	3.7	32.50
25427	SLE	**26.2**	**200**
25429	SLE	2.4	40.6
25431	SLE	2.0	27.2
25433	SLE	**179.0**	**200**
25434	SLE	3.4	26.4
25435	SLE	6.5	41.7
25437	SLE	2.6	64.7
25441	SLE	**40.4**	**200**
25442	SLE	3.0	37.8
25443	SLE	3.8	51.9
25444	SLE	4.3	59.6
25449	SLE	4.0	78.1
25450	SLE	**18.5**	**200**
25458	SLE	7.1	79.4
25459	SLE	6.1	29.6
25461	SLE	**17.0**	**171.0**
*25514*	SLE	**529**	**200**
25469	RA	0.1	31.7
25470	RA	7.3	70.8
25475	RA	0.0	32.0
25479	RA	0.1	37.7
25494	RA	2.4	28.7
25495	RA	1.2	29.7
25501	RA	2.4	51.1
25510	RA	3.4	29.2
25513	PM/Scl	3.1	43.8
25516	MCTD	3.8	32.3
25517	PM/Scl	1.3	94.9
25528	Scl	3.3	30.5
25529	Overlap syndrome	4.1	63.4
25530	Overlap syndrome	7.2	57.4
25536	Scl	3.2	80.3
25537	MCTD	3.5	29.7
25539	MCTD	1.5	26.6
25540	MCTD	4.5	33.5
25544	Overlap syndrome	8.8	99.7
25552	Overlap syndrome	3.4	32.2
25553	PM/Scl	9.6	41.4
25556	MCTD	2.8	34.3

Data in bold show positive samples in the SmD3 ELISA. MCTD = mixed connective tissue disease; PM/Scl = polymyositis scleroderma overlap syndrome; RA = rheumatoid arthritis; Scl = scleroderma; SLE = systemic lupus erythematosus

The newly defined cut-off value (100 units) was validated in a second, independent cohort of patients (panel III). At a cut-off of 25 units, 16 control samples and 47 SLE samples were positive, resulting in sensitivity of 47.0% and specificity of 84.0%. In contrast, when the new cut-off value was used, the sensitivity and specificity for SLE were 21.0 and 1000%, respectively (Table 1).

### Technical evaluation of the SmD peptide assays

**Figure 2:**
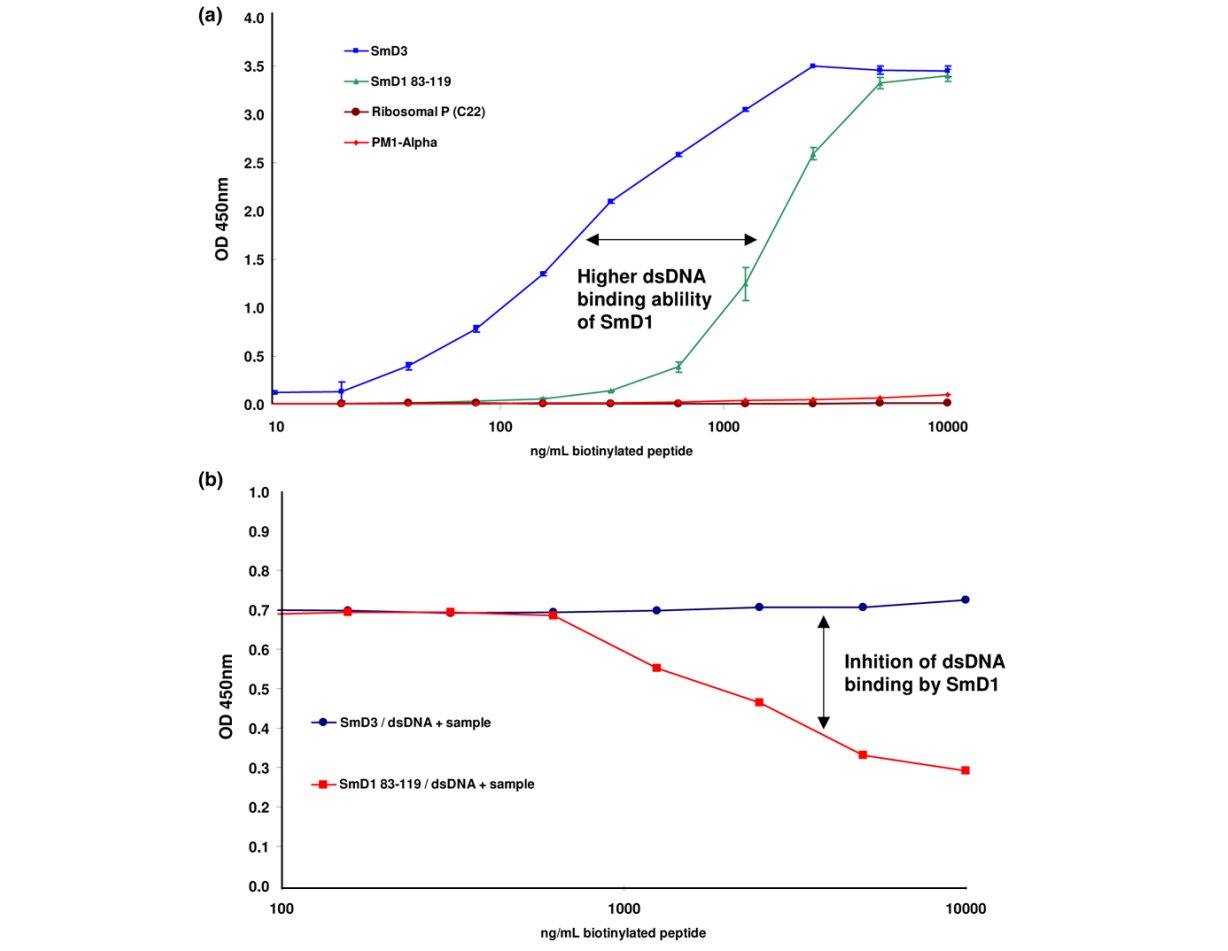
Binding of SmD peptides to dsDNA. **(a) **SmD1, SmD3 and biotinylated control peptides (PM1-α and Ribosomal P) were serially diluted in dilution buffer (0.01–10 μg/ml) and incubated on dsDNA-coated ELISA plates. Unbound peptides were removed by washing. Immobilized peptides were detected by streptavidin–horseradish peroxidase conjugate in combination with 3,3',5,5'-tetramethylbenzidine substrate. The SmD1 peptide showed dsDNA binding starting with a concentration of approximately 40 ng/ml, and the SmD3 peptide starting with approximately 0.6 μg/ml. No dsDNA binding could be observed with PM1-α and Ribosomal P. **(b) **The inhibitory effect of the SmD1 and SmD3 peptides was analysed by testing an anti-dsDNA-positive sample from a systemic lupus erythematosus patient on dsDNA-coated microtitre strips preincubated with increasing concentrations of the SmD peptides as described above. Detection of bound human dsDNA antibodies was according to the instructions for use of the dsDNA ELISA (Dr Fooke Laboratories). No inhibition was observed for SmD3, but inhibition was observed for SmD1 starting at a concentration of approximately 1 μg/ml. OD, optical density.

A panel of sera (*n *= 65) with anti-Sm reactivity, selected based on the test results of the Sm antibody assay contained in the addressable laser bead assay, was tested for anti-SmD1 and anti-SmD3 antibodies by ELISA. At the cut-off value (25 units) recommended by the manufacturer, 56/65 (86.2%) sera had a positive test result in the SmD1 ELISA. When the more specific cut-off value of 100 units was used, 38/65 (58.5%) samples showed anti-SmD1 reactivity (see Figure 2). Further, 34/65 (52.3%) of the sera tested positive for SmD3 antibodies at a cut-off value of 15 units as recommended by the manufacturer. When the borderline specimens (cut-off value 10 U/ml) were included, 38/65 (58.5%) samples were positive for anti-SmD3.

Analysis for agreement between both SmD ELISAs revealed concordance values between 66.2% (*P *= 0.0025; χ^2 ^= 9.15) and 90.8% (*P *< 0.0001; χ^2 ^= 39.37) depending on the cut-off values (see Figure 2). The CDC international reference serum panel was tested for autoantibodies to the SmD1 and SmD3 peptides. Samples 1 and 5 were positive for anti-SmD1 antibodies, and sample 5 was positive for anti-SmD3 antibodies. Sample 1 was borderline positive for anti-SmD3 antibodies (Table 3).

**Table 3:** Results of the Centre of Disease Control and Prevention antinuclear antibodies reference sera

Sample	SmD3	SmD1	RNP/Sm ELISA	SmD ELISA	Autoantibody [24, 35]
1	10.3	**115.6**	**3.0**	**1.9**	dsDNA, ssDNA, histone, (weak) Sm
2	1.4	11.4	0.3	0.3	(weak) SS-A, SS-B
3	1.9	18.1	**7.3**	**1.6**	(weak) Sm, SS-A, SS-B
4	2.5	19.6	**7.5**	0.4	U1 RNP
5	**>100.0**	**>200**	**7.8**	**6.0**	Histone, Sm
6	3.1	14.5	0.5	0.3	Nucleolar
7	0.0	7.7	0.2	0.3	SS-A
8	0.1	6	0.2	0.3	Centromere
9	1.7	18	0.2	0.3	Scl-70
10	0.5	3.1	0.1	0.1	Jo-1
11	0.6	11.9	0.3	0.4	PM/Scl complex

Bold data indicate positive test result in the respective test. PM/Scl complex = polymyositis/scleroderma overlap complex

### Relationship between anti-SmD peptide and anti-dsDNA reactivity

Thirty-six out of 65 (55.4%) of the anti-Sm-positive samples were also positive for anti-dsDNA antibodies by ELISA. Anti-dsDNA reactivity was confirmed in 19/36 (52.8%) anti-dsDNA ELISA-positive samples by indirect immunofluorescence on *C. luciliae *substrates. Two samples were positive for anti-dsDNA by ELISA and *C. luciliae *but negative for antibodies to both SmD peptides (see Table 4).

**Table 4:** Reactivity profile of Sm-positive/dsDNA-positive sera

Sample ID	ALBIA^a^		ELISA				ELISA dsDNA	*Crithidia luciliae *dsDNA
			
	RNP	Sm	RNP/Sm	SmD	SmD1	SmD3		
25480	435	396	9.2	4.8	189.1	11.2	3.9	Positive
25719	217	212	2.1	0.9	188.9	4.4	5.1	Positive
25794	487	1126	7.4	5.6	192.0	7.7	1.8	Positive
26301	167	330	6.1	1.2	183.2	>100.0	2.1	Positive
26488	126	127	1.0	0.6	17.2^b^	0.5^b^	1.8	Positive
27224	265	607	7.8	4.5	190.1	88.2	2.9	Positive
27960	165	326	7.8	5.7	182.5	23.2	3.3	Positive
28077	250	527	9.0	5.6	182.0	73.7	4.8	Positive
28242	65	102	4.0	1.6	71.2	0.7	11.1	Positive
28746	143	169	4.3	2.6	181.3	1.1	9.2	Positive
29236	98	164	5.9	1.7	182	264	6.8	Positive
29354	450	225	4.0	2.1	32.4	4.2	12.1	Positive
29659	161	132	4.9	2.2	30.1	3.3	11.6	Positive
29861	496	205	8.9	4.5	19.2^b^	3.3^b^	1.6	Positive
29907	538	520	9.3	6.2	185.0	243.0	12.2	Positive
30015	376	632	9.6	7.1	184.3	>100.0	5.0	Positive
31349	83	149	8.2	4.2	193.5	46.8	10.7	Positive
34249	348	140	8.1	4.6	>200.0	13.4	5.1	Positive
35784	494	553	8.9	5.6	>200.0	>100.0	8.3	Positive
Number/number positive	19/19	19/19	18/19	16/19	14/19	9/19	19/19	19/19

^a^The sample value in luminescence units(LU) can be classified as: negative, <20 LU; weak positive, 20–49 LU; moderate positive, 50–100 LU; strong positive, >100 LU.^b^dsDNA-positive/SmD-negative samples. ALBIA = Addressable laser bead assay.

Depending on the cut-off value, the concordance between anti-SmD1 and anti-dsDNA reactivity ranged from 60.0% (χ^2 ^= 1.05, *P *= 0.3056) to 69.2% (χ^2 ^= 3.02, *P *= 0.0820), and the concordance between anti-SmD3 and anti-dsDNA ranged from 53.9% (χ^2 ^= 0.08, *P *= 0.7828) to 60.0% (χ^2 ^= 1.05, *P *= 0.3056).

### Binding of SmD peptides to dsDNA

**Figure 3:**
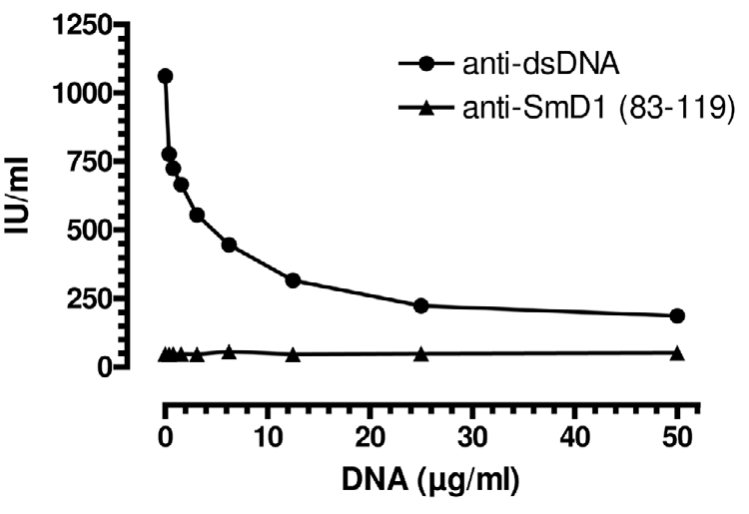
Bridging experiment. A serum sample with high-titre anti-dsDNA antibodies but no anti-SmD1 (amino acids 89–119) reactivity was spiked with increasing concentrations of dsDNA (plasmid, also used in dsDNA ELISA; Dr Fooke Laboratories) and incubated for 30 minutes at room temperature. The dilution series was subsequently tested for anti-dsDNA and anti-SmD1 reactivity in ELISA according to the instructions for use of the respective kit. The anti-dsDNA reactivity as determined by ELISA significantly decreased with increasing concentrations of dsDNA. No decrease in anti-SmD1 reactivity could be observed (data not shown). Error bars indicate the *x*-fold standard deviation of the duplicate determinations.

Binding of SmD-derived peptide to dsDNA was studied using dsDNA-coated ELISA plates (kit component of dsDNA ELISA, catalogue number 25004; Dr. Fooke Laboratories). Although both SmD peptides demonstrated binding to dsDNA, the binding of SmD1 was significantly higher than that of SmD3 (see Figure 3). No binding was observed with negative control peptides (PM1-α and Ribosomal P).

A spiking experiment was carried out to investigate a putative bridging effect of dsDNA on the reactivity of SmD1-negative samples (amino acids 83–119). Increasing concentrations of dsDNA (recombinant plasmid) showed an inhibitory effect on the binding of anti-dsDNA antibodies to dsDNA in the ELISA. No altered reactivity was observed in the SmD1 ELISA (see Figure 3). When an anti-dsDNA positive serum was tested for anti-dsDNA binding with increasing SmD1 and SmD3 peptide concentrations, no inhibition was observed for SmD3 but there was inhibition for SmD1 starting at a concentration of approximately 1 μg/ml (see Figure 3).

### Inhibition of anti-SmD3 reactivity with SmD1-derived peptide

Serum samples that have previously been identified as anti-SmD1-positive/SmD3-positive were diluted in sample buffer and were preincubated with increasing concentrations of SmD1 or SmD3 peptide, and the inhibition effect was determined. After preincubation with 100 μg/ml SmD1, anti-SmD3 reactivity was significantly inhibited (60%) in one out of four sera (data not shown).

## Discussion

Since the seminal identification of anti-Sm antibodies by Tan and Kunkel in 1966 [28], various techniques and different antigens have been used for the detection of Sm antibodies. These include double immunodiffusion, immunoblotting, immunoprecipitation, ELISAs, and multiplex assays using native antigens from different sources, purified or recombinant proteins, and synthetic peptides [3,14,15,28-31]. Recombinant SmBB' from bacteria or insect cells and native purified Sm antigen have also been used in kit development. Both of these antigens contain the cross-reacting epitope PPPGMRPP, which is present in SmBB' and in the U1-specific RNPs [7]. Since this epitope is frequently targeted by antibodies in sera from patients with various autoimmune diseases, most anti-Sm antibody assays with purified Sm or recombinant SmBB' fail to differentiate between SLE patients and patients with other autoimmune conditions. In a recent study we showed that the differentiation between the closely related autoimmune disorders SLE and MCTD can be improved by use of the SmD3 peptide ELISA [16].

In the present study, we analysed two SmD peptide ELISAs. Using the cut-off value recommended by the manufacturer for human sera (25 units), we confirmed the high sensitivity (70%/36%) and moderate specificity (91.7%/97.2%) of the SmD1 peptide-based assay as previously reported [15,20]; in our patient cohort, we found a sensitivity of 45.8% and a specificity of 77.8% (see Table 1). After receiver-operating characteristic analysis, we adjusted the cut-off value to 100 units for the SmD1 assay to achieve 100% specificity. Using this cut-off value we found the same patients positive for anti-SmD antibodies as with the SmD3 peptide assay, resulting in a sensitivity of 12.5% and agreement between the two tests of 100% (*P *< 0.0001; χ^2 ^= 127.61). It is noteworthy that anti-Sm antibodies are considered a highly specific, but only a modestly sensitive, marker for SLE. The discrepancy between this knowledge and the reported sensitivity and specificity of the SmD1 ELISA is critical in commercial laboratories that are not particularly interested in rheumatic disease serology. In those cases, general practitioners and rheumatologists often receive positive anti-Sm test reports without knowing which anti-Sm assay was used. With the expectation that anti-Sm is highly SLE specific, the clinician may arrive at a wrong decision about the diagnosis of the patient and commence inappropriate therapy.

In a second independent cohort of patients (panel III), the newly defined cut-off limit (100 units) of the SmD1 ELISA was validated. Using the new cut-off value, the high specificity (100.0%) and moderate sensitivity (21.0%) known for anti-Sm antibodies were confirmed. We therefore conclude that the reported difference in the assay performance between the two SmD peptide assays is mainly attributed to the different definitions of the cut-off. Since our patient cohort had been previously tested for anti-Sm antibodies using purified Sm antigens in other immunoassays, a direct comparison of the results is possible. Commercial immunoassays based on purified native Sm antigen demonstrate similar sensitivities of 10–12% but lower specificities of 88–94% when compared with the SmD peptide-based ELISAs [16]. The antigen employed in the addressable laser bead assay is also a conventionally purified Sm antigen comprising all Sm polypeptides, and may even contain low concentrations of other proteins such as U1-specific RNPs. These assays therefore detect a heterogeneous autoantibody population. In contrast, the SmD1 ELISA and the SmD3 ELISA are based on single peptides derived from the SmD sequence [14,15]. Consequently, when the peptide-based assays are used, only a subset of anti-Sm antibodies is detected.

Other Sm autoantibody specificities such as the cross-reactive antibodies recognizing the MCTD-specific epitope PPPGMRPP, which is shared between SmBB' and U1-specific RNPs, are not detected [7]. This explains the observation that not all anti-Sm-positive samples from the second serum panel (*n *= 65) were detected by the peptide-based immunoassays. Although anti-SmD peptide antibodies represent only a minor subpopulation of anti-Sm antibodies, based on the high sensitivity and specificity percentages as well as the observation that anti-SmD peptide antibodies can be used to discriminate MCTD from SLE patients, we conclude that these subpopulations represent important SLE-specific antibodies [14,15]. A mixture of RNP/Sm therefore represents an accurate tool to screen for anti-RNP/Sm antibodies, and synthetic SmD peptides are useful to determine the fine specificity of the patient samples.

Since the anti-SmD peptide ELISAs showed a high degree of concordance, we performed a competitive ELISA to study the putative cross-reactivity. One out of four sera had a significant decrement reactivity to SmD3 when preincubated with SmD1. We therefore conclude that some patients produce autoantibodies that cross-react with SmD1 and SmD3.

Riemkasten and colleagues reported anti-SmD reactivity in 70.0% of SLE patients and in only 8.3% of controls using a SmD1 synthetic peptide [15]. This peptide, but not the full-length protein, has been shown to bind dsDNA contained in blocking reagents, which may result in the detection of anti-dsDNA antibodies in the SmD peptide ELISA [32]. In the present study we confirmed the dsDNA binding property of the SmD1 peptide. This finding was further supported by the inhibition of dsDNA binding of human anti-dsDNA antibodies from a SLE patient. Based on this observation, one might speculate that all sera with high titres of anti-dsDNA antibodies will also be positive in the anti-SmD1 ELISA. We found highly positive anti-dsDNA sera, however, which were negative for anti-SmD1 in the ELISA. Furthermore, no increase in anti-SmD1 reactivity could be induced by increasing the concentrations of dsDNA. Coincident reactivity with dsDNA and different Sm antigens, including full-length native antigens and SmD-derived peptides, has been reported by several authors [33,34]. Although correlation of anti-dsDNA and anti-SmD1 reactivity (*P *= 0.0058) and of anti-dsDNA and anti-SmD3 reactivity (*P *< 0.001) was found in previous studies [14,15], we could not confirm such a correlation in this study. This might be explained by the different practices of patient selection. While the previous studies used unselected SLE patients to establish the relationship between anti-dsDNA and anti-SmD, in the current investigation a panel of sera was selected based on the presence of anti-Sm antibodies. The lack of concordance between anti-dsDNA and anti-SmD peptide reactivity provides additional evidence against the hypothesis that anti-dsDNA antibodies are detected by the SmD1 (amino acids 83–119) ELISA.

In a previous study, autoantibodies to various autoantigens in the CDC reference sera were studied using different technologies, including the immunoblot method [35]. Only sample AF/CDC5 showed bands corresponding to the multiple bands of the Sm complex. All other samples were negative for anti-Sm antibodies by various techniques [35]. There is a pressing need, however, for the characterization of this reference panel using newer technologies for the detection of autoantibodies. We therefore tested the entire CDC reference sera panel for SmD peptide reactivity. The apparent discrepant result of the AF/CDC1 sample may be explained by low titres of anti-SmD antibodies present in this serum. A previous study has also reported discrepant results for this serum sample [36].

The SmD1, SmD3 and SmBB' polypeptides have recently been shown to contain sDMA, and this constitutes a major autoepitope within the C-terminus of SmD1 and SmD3 [14,18,37]. In one of these studies, a synthetic peptide of SmD1 (amino acids 95–119) containing sDMA demonstrated significantly increased immunoreactivity compared with the nonmodified peptide [18]. The new SmD3 assay is also based on Sm peptide containing sDMA [14]. Since no study has been published that describes the cloning, expression and purification of SmD1/D3 or SmBB' containing sDMA, either highly purified native SmD or synthetic Sm peptides should be used as antigens to detect anti-Sm antibodies in the diagnosis of SLE. Whether this modified amino acid also plays a central role in the development of the SLE-specific B-cell immune response to the Sm particles remains a matter of speculation.

Synthetic peptides represent ideal antigenic targets for immunoassays because they can easily be produced in high quality and in quantities with low lot-to-lot variations. In 1998 Schellekens and colleagues described the identification of a citrullinated cyclic peptide that has become an important and reliable marker for the diagnosis of rheumatoid arthritis [38]. Today's sophisticated epitope mapping methods will probably lead to the identification of additional peptides, which can be used as specific targets in diagnostic and therapeutic approaches to patient management. This may lead to a new scientific research area in peptide engineering with high potential for the development of novel diagnostic and therapeutic products. The identification of more peptides clearly defined by their amino acid sequence that are autoantibody targets may accelerate progress in the international standardization of the autoantibody test, an elusive goal which has been pursued for more than 20 years.

## Conclusion

In the present study we have analysed two anti-Sm antibodies assays using synthetic SmD-derived peptides. In summary, we have found that the previously reported difference in the sensitivity and specificity of both tests is caused by the cut-off definition. After adjustment of the cut-off value of the SmD1 peptide assay to 100 units we found excellent agreement (*P *< 0.0001) between the two assays, with the same sensitivity (12.5%) and disease specificity (100%). Moreover, we have shown that the high binding properties of SmD1 (amino acids 83–119) to dsDNA have no significant effect on the diagnostic accuracy of the SmD1 ELISA. Based on these findings, we conclude that both SmD peptide-based assays represent a reliable tool for the highly specific detection of anti-Sm antibodies, and that SmD-derived peptides may become the gold standard for the detection of anti-Sm antibodies.

## Abbreviations

CDC = Centre for Disease Control and Prevention; dsDNA = double-stranded DNA; ELISA = enzyme linked immunosorbent assay; MCTD = mixed connective tissue disease; RNP = ribonucleoprotein; sDMA = symmetrical dimethylarginine; SLE = systemic lupus erythematosus.

## Competing interests

MM was employed at Phadia GmbH (Freiburg, Germany) and received financial compensation for the development of the SmD3 peptide assay. Now, M. Mahler is employee of Dr. Fooke Laboratories which sell an Sm ELISA used in this publication. FH is one of the inventors of the SmD1 peptide assay and received payments based on the turnover of the ELISA from Human (formerly Imtec, Berlin). MJF is a paid consultant of ImmunoConcepts (Sacramento, CA, US).

## Authors' contributions

MM planned and conducted the study and filed the manuscript. MJF provided clinically defined serum samples, performed the addressable laser bead immunoassays, consulted in the evaluation of the data and helped to write the manuscript. AW performed the ELISAexperiments and helped with data analysis. FH consulted in the evaluation of the data and helped to write the manuscript. All authors read and approved the final manuscript.
